# The interplay between bicarbonate kinetics and gastrointestinal upset on ergogenic potential after sodium bicarbonate intake: a randomized double-blind placebo-controlled trial

**DOI:** 10.1038/s41598-023-34343-0

**Published:** 2023-05-01

**Authors:** Krzysztof Durkalec-Michalski, Paulina M. Nowaczyk, Joanna Kamińska, Bryan Saunders, Igor Łoniewski, Dominika Czubaszek, Michal Steffl, Tomasz Podgórski

**Affiliations:** 1 Department of Sports Dietetics, Poznan University of Physical Education, 61-871 Poznan, Poland; 2 Sport Sciences-Biomedical Department, Faculty of Physical Education and Sport, Charles University, Prague, Czech Republic; 3 Department of Physiology and Biochemistry, Poznan University of Physical Education, Poznan, Poland; 4 Applied Physiology and Nutrition Research Group, School of Physical Education and Sport, Rheumatology Division, Faculdade de Medicina FMUSP, University of São Paulo, São Paulo, Brazil; 5 Institute of Orthopedics and Traumatology, Faculty of Medicine FMUSP, University of São Paulo, São Paulo, Brazil; 6 Department of Biochemical Sciences, Pomeranian Medical University in Szczecin, Szczecin, Poland

**Keywords:** Biochemistry, Physiology

## Abstract

This double-blind placebo-controlled cross-over study utilized comprehensive monitoring of blood bicarbonate (HCO_3_^¯^) kinetics and evaluation of gastrointestinal (GI) upset to determine their impact on an ergogenic potential of sodium bicarbonate (SB) co-ingested with carbohydrate (CHO). Nineteen CrossFit athletes performed 6 bouts of 15 s Wingate Anaerobic Test (WAnT) 90 min post-ingestion of 0.4 g·kg^−1^ body mass (BM) of SB (SB + CHO treatment) or PLA (PLA + CHO treatment) with 15 g CHO. Blood HCO_3_^¯^ concentration was evaluated at baseline, 30-, 60-, 75- and 90 min post-ingestion, in between WAnT bouts, and 3 and 45 min post-exercise, while GI upset at 120 min after protocol started. Control (no supplementation; CTRL) procedures were also performed. An effective elevation of extra-cellular buffering capacity was observed 60–90 min post-ingestion of SB + CHO. At mean peak blood HCO_3_^¯^, or at start of exercise an increase > 6 mmol·L^−1^ in HCO_3_^¯^ was noted in 84% and 52.6% participants, respectively. SB + CHO did not prevent performance decrements in WAnT bouts. There were no significant relationships between changes in blood HCO_3_^¯^ and WAnTs’ performance. Total GI was significantly higher in SB + CHO compared to CTRL, and stomach problems in SB + CHO compared to CTRL and PLA + CHO. There were inverse associations between peak- (*p* = 0.031; *r* = − 0.495), average- (*p* = 0.002; *r* = − 0.674) and minimum power (*p* = 0.008; *r* = − 0.585) and total GI upset, as well as average power and severe GI distress (*p* = 0.042; *r* = − 0.471) at SB + CHO. The implemented dose of SB + CHO was effective in improving buffering capacity, but did not prevent decrements in WAnTs’ performance. GI side effects were crucial in affecting the ergogenic potential of SB and thus must be insightfully monitored in future studies.

## Introduction

High-intensity functional training (HIFT) is derived from (1) well-studied high-intensity interval training (HIIT), in which relatively short bouts of high-intensity exercise separated by a recovery using low-intensity or inactivity are used as an alternative to traditional aerobic training to promote metabolic conditioning^1,2^, and (2) strength training paradigms^3^. HIFT training stresses both aerobic and anaerobic energy pathways, and develops power, strength, flexibility, speed, endurance, agility and coordination^1^. HIFT is executed at a high intensity and emphasizes functional, multi-joint movements via endurance, strength, power and speed stimulating exercises^2,4^.

The majority of studies focusing on HIFT methodology have used a cross-training template^4,5^. Cross-training’s specific modes of exercise (such as CrossFit’s programs) include power/Olympic lifting (i.e., squats, cleans, deadlifts, bench press, and push-presses), gymnastics (i.e., pull-ups, lunges, knees to elbows, handstand push-ups, push-ups, and sit-ups), and aerobic exercise/metabolic conditioning (i.e., swimming, running, and rowing). These high intensity exercise movements are often performed for specific lengths of time with little or no rest^6^.

Despite the growing popularity of HIFT and CrossFit training, limited interventional data exist on dietary and supplementation strategies to enhance performance in CrossFit^7,8^. So far, there are only two studies investigating the effectiveness of sodium bicarbonate (SB)—which in fact is one of the most widely studied ergogenic aids—on CrossFit-like performance^9,10^. According to the latest International Society of Sport Nutrition (ISSN) position stand^11^, supplementation with SB (doses from 0.2 to 0.5 g·kg^−1^ body mass < BM >) improves performance in muscular activities, various combat sports (e.g., boxing, judo, karate, taekwondo, wrestling), and in high-intensity cycling, running, swimming and rowing. The ergogenic effects of SB are mostly established for high-intensity exercise tasks that last between 30 s and 12 min^11^. With the latter in mind, SB may exert great ergogenic potential in HIFT such as CrossFit. During short-term high-intensity exercise, the intramuscular ATP demands exceed the maximum rate of ATP resynthesis by mitochondria. In these circumstances, ATP production heavily relies on anaerobic systems—phosphorylcreatine (ATP-PCr) hydrolysis or glycolysis. Intense exercises that are more reliant on glycolysis (i.e., those lasting from ~ 30 s to ~ 5 min) results in greater H^+^ generation^11^. Accumulation of H^+^ (leading to muscle acidosis and a decrease in muscle pH) has been shown to be an important contributor to fatigue due to its inhibitory effects on key glycolytic enzymes^11^, depressing Ca^2+^ sensitivity, as well as direct effects on cross-bridge cycling^11^. SB ingestion elicits ergogenic effect by increasing the concentration of blood bicarbonate, a buffer that contribute to maintain extra- and (indirectly) intracellular pH^11^. In the stomach, SB rapidly reacts with hydrochloric acid to form NaCl, CO_2_, and H_2_O; excess bicarbonate that does not neutralize gastric acid rapidly empties into the small intestine and is absorbed^12^. The reaction of NaHCO_3_ and HCl in gastric lumen increases the pH of the gastric lumen, which stimulates gastric parietal cells to secrete more HCl into the gastric lumen, thus resulting in the extrusion of HCO_3_¯ into the perigastric capillaries and eventually into the systemic circulation (one HCO_3_¯ ion on each H^+^ ion used in the reaction in the gastric lumen). The surplus HCO_3_¯ is eliminated via the kidney^13^ and carbon dioxide formed during this process is eliminated via the lungs^14^.

Numerous different protocols of SB supplementation have been investigated, however, so far only two protocols have been implemented in CrossFit-trained athletes^9,10^. One^9^ was a 10 day progressive-dose regime implemented in male and female CrossFit practitioners. The protocol resulted in overall improvement in discipline-specific performance (as measured during *Fight Gone Bed* test) by ~ 6.1% and enhancement in selected indices of aerobic capacity (as measured during incremental cycling). The chronic progressive supplementation regime prevented the occurrence of gastrointestinal (GI) side effects. The second dosing protocol^10^, was acute supplementation of 0.3 g·kg^−1^_BM_ of SB ingested in three doses with 10 min intervals between doses, with the last dose ingested 60 min before exercise. This protocol neither improved discipline specific performance (as measured during ‘*Cindy*’ workout), nor altered hemodynamic and perceptual parameters. Moreover, no GI side effects were observed.

Although the performance benefits with SB are well documented, data linking the kinetics of blood HCO_3_^¯^, GI occurrence and anaerobic performance after ingestion of high doses of SB in conjunction with carbohydrate (CHO) in HIFT-trained athletes is lacking. Thus, it was hypothesized that the ingestion of 0.4 g·kg^−1^_BM_ of SB + 15 g CHO ninety min before repeated-bouts of highly intensive exercise would result in the elevation of blood HCO_3_^¯^ above the almost certain ergogenic threshold (> 6 mmol·L^−1^)^15,16^ and minimize decrements of performance in consecutive bouts of the WAnT in CrossFit-trained athletes. It was also assumed that the conjunction of SB with small dose of CHO would minimize GI side effects.

## Results

All the performed and presented results’ analyses of both primary and secondary outcomes covered 19 participants and the analyses were performed according to originally assigned groups.

### Blood sample analysis

**Figure 1:**
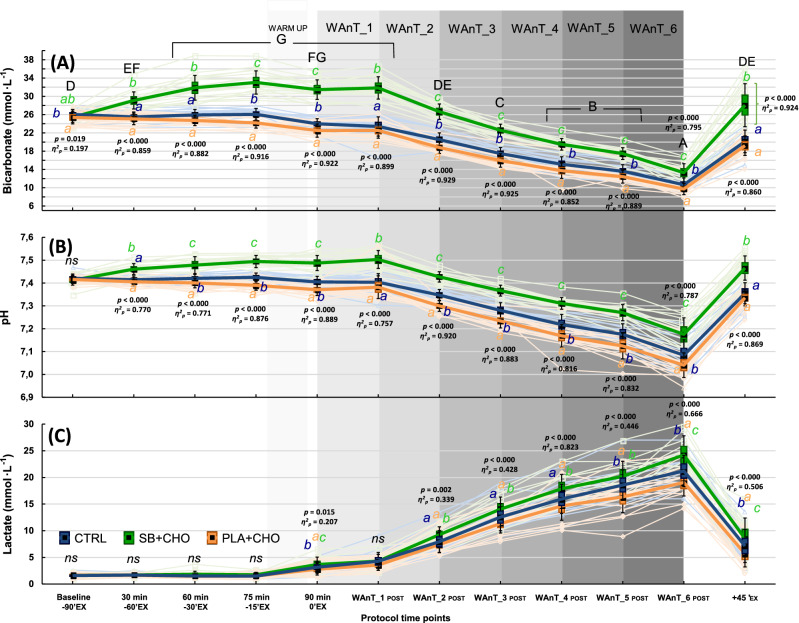
Mean and individual (light-colour lines) blood bicarbonate (**A**), pH (**B**), lactate (**C**) values. Data are expressed as means (square), 95% CI (box), 95% CI + SD (whisker), raw data (light-colour lines). ^a,b,c^Different lowercased letters refers to significant differences between study conditions (CTRL, SB + CHO, PLA + CHO); ^A–G^different uppercased letters refers to significant differences between measuring time points (from − 90′_EX_ to + 45′_EX_) within SB + CHO condition.

The intake of 0.4 g·kg^−1^_BM_ of SB + 15 g CHO (SB + CHO) resulted in a mean peak of blood HCO_3_^¯^ (33.0 ± 2.5 mmol·L^−1^) 75 min post-ingestion (Fig. 1A), which was 15 min before the test exercises started (− 15′_EX_). As many as 79% of participants reached peak HCO_3_^¯^at this time point, while for the remaining participants, the pre-exercise peak HCO_3_^¯^ occurred between 60 < 5% > and 90 < 16% > min after ingestion, which was − 30′_EX_ and 0′_EX_. The HCO_3_^¯^at CTRL and PLA + CHO at 75 min post-ingestion (− 15′_EX_) were 26.1 mmol·L^−1^ and 24.2 mmol·L^−1^ (substantially lower compared to SB + CHO and significantly different between PLA + CHO vs CTRL), and were not different from the corresponding pre-ingestion values (− 90′_EX_; CTRL: 26.0 mmol·L^−1^ and PLA + CHO: 25.4 mmol·L^−1^). HCO_3_^¯^ was clearly and effectively elevated after SB + CHO ingestion. Except the baseline measurement (− 90′_EX_), mean HCO_3_^¯^ at each measuring point at PLA + CHO and at CTRL were substantially lower compared to SB + CHO. Concentration of HCO_3_^¯^ at − 15′_EX_, − 0′_EX_ and from WAnT_2_POST_ to WAnT_6_POST_ were significantly lower at PLA + CHO compared to CTRL.

**Figure 2:**
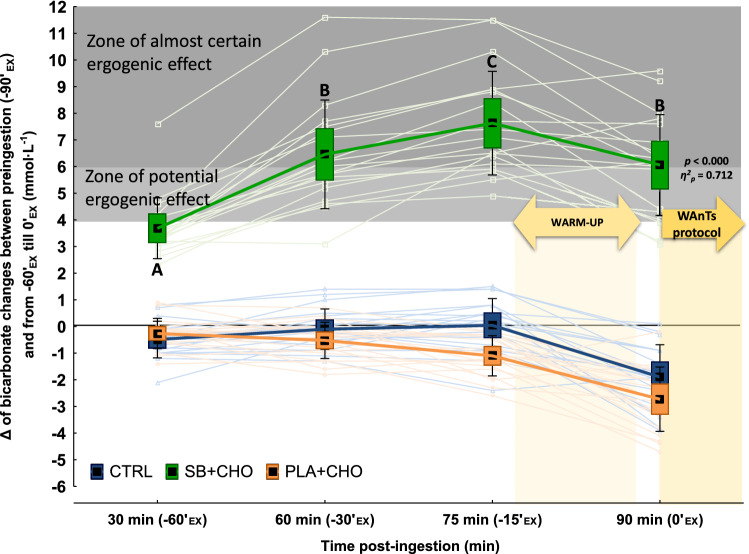
∆ of mean and individual (light-colour line) bicarbonate changes between baseline (pre-ingestion) and 30, 60, 75 and 90 min after SB + CHO or PLA + CHO intake or the first blood drawn (CTRL). Data are expressed as means (square), 95% CI (box), 95%CI + SD (whisker), raw data (light-colour lines). ^A–C^Different uppercased letters refers to significant differences between measuring time points (from − 90′_EX_ to + 45′_EX_) within SB + CHO condition.

The mean peak change (increase) in HCO_3_^¯^ from baseline was + 7.8 ± 1.9 mmol·L^−1^ and was observed 75 min after ingestion of SB + CHO (− 15′_EX_; and 2 min before warm-up) (Fig. 2). Approximately 16% of participants’ peak increase in HCO_3_^¯^ was within the range 4–6 mmol·L^−1^ and 84% of participants had increases of > 6 mmol·L^−1^. The mean increase of HCO_3_^¯^ at 0′_EX_ (90 min post-ingestion) was + 6.1 ± 1.9 mmol·L^−1^. In 9 participants (47.4%) the increase in HCO_3_^¯^ at this time point fell within range 4–6 mmol·L^−1^ and in the remaining 10 participants (52.6%) it was > 6 mmol·L^−1^. The increase in HCO_3_^¯^ at − 15′_EX_ was substantially higher compared to remaining time points, and at − 30′_EX_ and − 15′_EX_ substantially higher compared to − 60′_EX._

There were no differences in baseline (pre-ingestion) pH values between study conditions (Fig. 1B). However, at each remaining time-point, the mean pH was substantially higher in SB + CHO compared to PLA + CHO and CTRL. Furthermore, at the points from − 30′_EX_ to 0′_EX_ and from WAnT_2_POST_ to WAnT_6_POST,_ pH was significantly higher in CTRL compared to PLA + CHO.

After SB + CHO ingestion the mean peak of blood pH (7.503 ± 0.039) was observed after the first bout of WAnT (WAnT_1_POST_; Fig. 1B), with ~ 42% of participants peaking at this point. The mean increase in pH at SB + CHO at WAnT_1_POST_ from baseline was + 0.091 ± 0.037 and the absolute differences in pH at this time point between SB + CHO vs CTRL and SB + CHO vs PLA + CHO were + 0.100 ± 0.045 and + 0.121 ± 0.059. Furthermore, the peak pH increase (based on individual blood pH peaks reached at the individual time points) was + 0.106 ± 0.027.

There were no differences in lactate concentration between study conditions from − 90′_EX_ to − 15′_EX_ and at WAnT_1_POST_ (Fig. 1C). At each remaining time point, lactate was higher after SB + CHO ingestion compared to lactate in PLA + CHO and CTRL (excluding WAnT_5_POST_). However, there were differences between CTRL and PLA + CHO at 0′_EX_, WAnT_5_POST_, WAnT_6_POST_ and + 45′_EX_ (recovery phase), with blood lactate higher in CTRL compared to PLA + CHO.

It should also be highlighted that at all time points after ingestion of SB + CHO compared to CTRL and PLA + CHO, the concentration of H^+^ was significantly lower, while base excess was significantly higher (Supplementary table S1). The results of remaining blood acid–base balance indices are given in Supplementary table S1.

### The WAnT bouts performance

**Figure 3:**
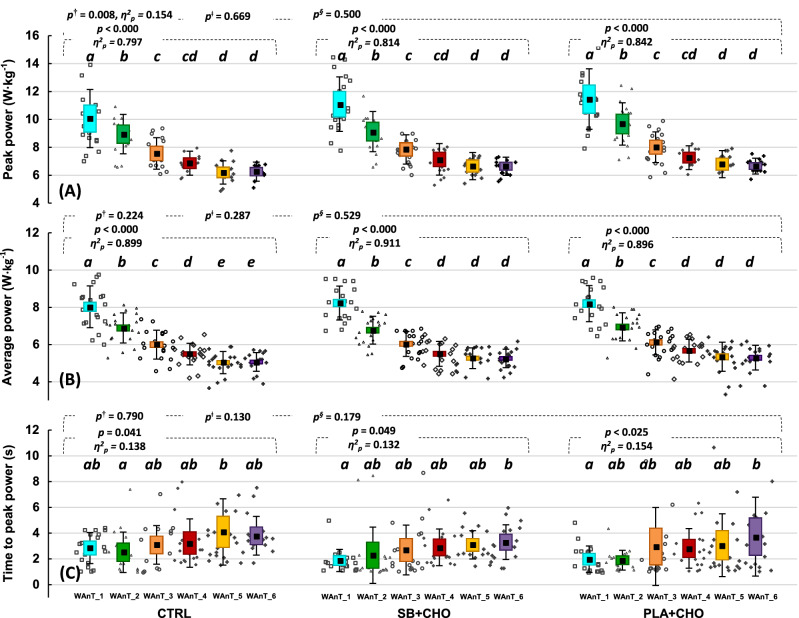
Mean peak power (**A**), average power (**B**), time to peak power (**C**) during consecutive bouts of 15-s WAnTs. Data are expressed as means (square), 95% CI (box), 95% CI + SD (whisker), raw data. ^a–e^Different lowercased letters refers to significant differences between the WAnT bouts (WAnT_1-WAnT_6); RM ANOVA models: ^†^bouts *x* treatment, ^ǂ^bouts *x* treatment *x* treatment order and ^§^bouts *x* treatment *x* gender).

Within each of the study conditions (CTRL, SB + CHO, PLA + CHO), performance during the WAnTs decreased gradually in consecutive bouts (Fig. 3A–C). The PP in WAnT_3 to WAnT_6 was significantly lower compared to WAnT_1 and WAnT_2 within each of study conditions (Fig. 3A). Regarding AP (Fig. 3B), there was a decline from WAnT_1 to WAnT_4 in CTRL, and from WAnT_1 to WAnT_3 in SB + CHO and PLA + CHO, and remained stable (but significantly lower from previous bouts) between WAnT_5 and WAnT_6 in CTRL, and between WAnT_4 and WAnT_6 for SB + CHO and PLA + CHO. Moreover, T_to_PP (Fig. 3C) significantly increased between WAnT_1 and WAnT_6 within the SB + CHO and PLA + CHO conditions. It was also significantly higher at WAnT_5 compared to WAnT_2 in CTRL.

There was no effect of treatment order on exercise performance (PP, AP, T_to_PP) in individual bouts (WAnT_1-WAnT_6) of WAnT.

There was a significant treatment *x* WAnT bout main effect for PP. However, the main effects were no longer significant when treatment order (WAnT bouts *x* treatment *x* treatment order) or gender (WAnT bouts *x* treatment *x* gender) were considered in the models (Fig. 3A). None of the examined main effects were significant with respect to AP (Fig. 3B) or T_to_PP (Fig. 3C) in any of the studied treatment conditions.

The mean PP of the 6 bouts of WAnT was significantly higher in SB + CHO and PLA + CHO compared to CTRL, and the main effect remained significant after including treatment order (Table 1), but not gender, in the model. Mean PD was significantly higher in SB + CHO and PLA + CHO compared to CTRL, but was no longer significant after considering treatment order or gender in the models. However, mean T_to_PP was significantly lower in SB + CHO compared to CTRL (but not PLA + CHO), and the main effect remained significant when considering treatment order in the model.

**Table 1:** Mean results from entire 6-bouts of 15-s WAnTs protocol.

	CTRL *n* = 19	SB + CHO *n* = 19	PLA + CHO *n* = 19	*p* *η*^2^_p_	*p*^†^ *η*^2^_p_	*p*^ǂ^ *η*^2^_p_
Peak power						
(W·kg^−1^)	7.65 ± 1.02^a^	8.09 ± 1.00^b^	8.31 ± 1.05^b^	0.000	0.033	0.242
(95% CI)	7.16–8.14	7.60–8.57	7.80–8.82	0.638	0.202	0.090
Average power						
(W·kg^−1^)	6.08 ± 0.66	6.18 ± 0.60	6.26 ± 0.66	0.061	0.222	0.482
(95% CI)	5.76–6.40	5.89–6.46	5.95–6.58	0.170	0.095	0.048
Minimum power						
(W·kg^−1^)	4.57 ± 0.54	4.54 ± 0.50	4.61 ± 0.54	0.604	0.307	0.496
(95% CI)	4.31–4.83	4.30–4.78	4.35–4.87	0.031	0.075	0.044
Power drop						
(W·kg^−1^)	3.09 ± 0.68^a^	3.55 ± 0.86^b^	3.70 ± 0.77^b^	0.000	0.552	0.760
(95% CI)	2.76–3.42	3.13–3.96	3.33–4.07	0.509	0.039	0.180
Time to peak power						
(s)	3.26 ± 1.09^b^	2.69 ± 0.87^a^	2.74 ± 1.55^ab^	0.023	0.002	0.099
(95% CI)	2.74–3.79	2.27–3.11	1.99–3.49	0.221	0.345	0.143

Data are mean ± SD, 95% CI. ^†^treatment *x* treatment order; ^ǂ^treatment *x* gender; ^a,b^different letters refers to significant differences between study conditions.

### Elevation of blood bicarbonate and anaerobic capacity

There were no significant linear relationships between changes in blood HCO_3_^¯^ from baseline to peak HCO_3_^¯^ concentration or changes from baseline and blood HCO_3_^¯^ at 0′_EX_, and PP, AP, MP, PD and T_to_PP in consecutive individual bouts of WAnT (Supplementary Table S2).

### Gastrointestinal side effects

Reporting of stomach problems was substantially higher in SB + CHO compared to CTRL and PLA + CHO (Table 2). Apart from that, there were no substantial differences in the intensification of any other of GI side effects between SB + CHO and PLA + CHO (Table 2). Symptoms such as flatulence, urge to defecate, belching or diarrhea, as well as total GI symptoms and less severe GI symptoms were higher after SB + CHO ingestion compared to CTRL.

**Table 2:** Gastrointestinal side effects.

		CTRL	SB + CHO	PLA + CHO
Stomach problems				
* p*	0.000	0.0 ± 0.5^a^	2.0 ± 3.0^b^	0.0 ± 2.0^a^
Kendall’s *W*	0.445	0.0–0.5	1.5–4.5	0.0–2.0
Nausea				
* p*	0.014	0.0 ± 1.5	1.5 ± 3.0	0.0 ± 2.0
Kendall’s *W*	0.213	0.0–1.5	0.0–3.0	0.0–2.0
Dizziness				
* p*	0.038	1.0 ± 3.0	1.5 ± 2.5	0.0 ± 1.5
Kendall’s *W*	0.164	0.0–3.0	0.0–2.5	0.0–1.5
Headache				
* p*	0.600	2.5 ± 4.0	2.0 ± 2.5	2.0 ± 3.0
Kendall’s *W*	0.025	0.0–4.0	1.0–3.5	0.0–3.0
Flatulence				
* p*	0.007	0.0 ± 0.5^a^	2.0 ± 2.5^b^	0.0 ± 2.0^ab^
Kendall’s *W*	0.249	0.0–0.5	0.5–3.0	0.0–2.0
Urge to urinate				
* p*	0.032	0.0 ± 0.0	0.0 ± 2.0	0.0 ± 0.5
Kendall’s *W*	0.172	0.0–0.0	0.0–2.0	0.0–0.5
Urge to defecate				
* p*	0.000	0.0 ± 0.0^a^	3.0 ± 5.0^b^	1.0 ± 2.5^ab^
Kendall’s *W*	0.435	0.0–0.0	0.0–5.0	0.0–2.5
Belching				
* p*	0.001	0.0 ± 0.5^a^	2.0 ± 2.0^b^	1.0 ± 1.0^ab^
Kendall’s *W*	0.346	0.0–0.5	0.0–2.0	1.0–2.0
Heartburn				
* p*	0.368	0.0 ± 0.0	0.0 ± 0.0	0.0 ± 0.0
Kendall’s *W*	0.050	0.0–0.0	0.0–0.0	0.0–0.0
Bloating				
* p*	0.046	0.0 ± 0.0	1.5 ± 2.5	0.0 ± 0.0
Kendall’s *W*	0.154	0.0–0.0	0.0–2.5	0.0–0.0
Stomach cramps				
* p*	0.485	0.0 ± 0.0	0.0 ± 1.0	0.0 ± 0.0
Kendall’s *W*	0.036	0.0–0.0	0.0–1.0	0.0–0.0
Intestinal cramps				
* p*	0.092	0.0 ± 0.0	0.0 ± 1.0	0.0 ± 0.0
Kendall’s *W*	0.120	0.0–0.0	0.0–1.0	0.0–0.0
Urge to vomit				
* p*	0.097	0.0 ± 0.0	0.5 ± 2.0	0.0 ± 1.0
Kendall’s *W*	0.117	0.0–0.0	0.0–2.0	0.0–1.0
Vomiting				
* p*	0.368	0.0 ± 0.0	0.0 ± 0.0	0.0 ± 0.0
Kendall’s *W*	0.050	0.0–0.0	0.0–0.0	0.0–0.0
Diarrhea				
* p*	0.000	0.0 ± 0.0^a^	4.0 ± 4.0^b^	1.0 ± 3.0^ab^
Kendall’s *W*	0.477	0.0–0.0	1.0–5.0	0.0–3.0
Side ache left				
* p*	0.607	0.0 ± 0.0	0.0 ± 0.0	0.0 ± 0.0
Kendall’s *W*	0.025	0.0–0.0	0.0–0.0	0.0–0.0
Side ache right				
* p*	0.097	0.0 ± 0.0	0.0 ± 0.0	0.0 ± 0.0
Kendall’s *W*	0.117	0.0–0.0	0.0–0.0	0.0–0.0
Muscle cramps				
* p*	0.168	0.0 ± 1.5	0.0 ± 0.0	0.0 ± 0.5
Kendall’s *W*	0.089	0.0–1.5	0.0–0.0	0.0–0.5
Cold shivering				
* p*	0.039	0.0 ± 0.0	0.0 ± 1.5	0.0 ± 0.0
Kendall’s *W*	0.162	0.0–0.0	0.0–1.5	0.0–0.0
Total GI symptoms				
* p*	0.000	4.0 ± 10.5^a^	26.0 ± 17.5^b^	11.0 ± 15.5^ab^
Kendall’s *W*	0.387	3.0–13.5	14.5–32.0	6.0–21.5
Severe GI symptoms				
* p*	0.819	0.0 ± 0.0	0.0 ± 0.0	0.0 ± 0.0
Kendall’s *W*	0.010	0.0–0.0	0.0–0.0	0.0–0.0
Less severe GI symptoms				
* p*	0.000	0.0 ± 4.0^a^	9.5 ± 5.5^b^	3.5 ± 5.5^ab^
Kendall’s *W*	0.469	0.0–4.0	5.5–11.0	2.0–7.5

Data are median ± IQR, upper quartile–lower quartile; ^a,b^different letters refers to significant differences between study conditions (CTRL, SB + CHO, PLA + CHO).

### Gastrointestinal side effects and anaerobic performance

There was an inverse relationship between mean PP, AP, MP and total GI symptoms in SB + CHO, and an inverse relation between MP and total GI symptoms in PLA + CHO (Table 3). The score of severe GI symptoms was inversely related to AP in SB + CHO and the score of less severe GI symptoms was positively correlated with T_to_PP in PLA + CHO.

**Table 3:** Regression between mean results from entire 6-bouts of 15-s WAnTs and GI symptoms.

	CTRL	SB + CHO	PLA + CHO	CTRL	SB + CHO	PLA + CHO	CTRL	SB + CHO	PLA + CHO
	Total GI symptoms score			Severe GI symptoms			Less severe GI symptoms		
Peak power									
* p*	0.863	**0.031**	0.112	0.861	0.107	0.220	0.422	0.900	0.246
* r*	0.042	**− 0.495**	**− **0.387	**− **0.043	**− **0.382	**− **0.304	0.196	**− **0.031	**− **0.288
Average power									
* p*	0.564	**0.002**	0.065	0.481	**0.042**	0.303	0.959	0.414	0.309
* r*	**− **0.141	**− 0.674**	**− **0.444	**− **0.172	**− 0.471**	**− **0.257	**− **0.013	**− **0.199	**− **0.254
Minimum power									
* p*	0.107	**0.008**	**0.034**	0.149	0.202	0.303	0.340	0.436	0.364
* r*	**− **0.381	**− 0.585**	**− 0.501**	**− **0.344	**− **0.306	**− **0.257	**− **0.232	**− **0.190	**− **0.228
Power drop									
* p*	0.207	0.349	0.410	0.149	0.394	0.516	0.076	0.894	0.184
* r*	0.303	**− **0.227	**− **0.207	0.344	**− **0.207	**− **0.163	0.417	0.033	**− **0.328
Time to peak power									
* p*	0.526	0.443	0.077	0.598	0.648	0.102	0.856	0.484	**0.026**
* r*	0.155	0.187	0.428	0.129	0.112	0.397	**− **0.045	0.171	**0.523**

Significant values are in [bold].

*GI* gastrointestinal.

## Discussion

In this study, mean peak blood bicarbonate (HCO_3_^¯^) occurred after 75 min following ingestion of SB + CHO, with 79% of participants peaking at this time point. There were clear decrements in performance during subsequent WAnTs, regardless of study condition. There was a significant main effect between conditions for PP, however it lost significance after including treatment order or gender in the models. Mean PP across the six WAnTs was higher with SB + CHO and PLA + CHO compared to CTRL, but there was no difference between SB + CHO and PLA + CHO. There were no associations between the level of elevation of blood HCO_3_^¯^ and WAnT performance. Severity of GI side effects were not significantly different between SB + CHO and PLA + CHO, but substantially higher compared to CTRL. However, there was an inverse interaction between GI side effects and performance indices (PP, AP, MP, PD) with SB + CHO, meaning GI worsened performance in WAnT.

In the current study after ingestion of 0.4 g·kg^−1^_BM_ SB combined with 15 g CHO an effective and desirable *plateau* in an increased HCO_3_^¯^ concentration was achieved and maintained from − 30′_EX_ (60 min post-ingestion) until WAnT_1_POST_, fluctuating between 31.4 and 33.0·mmol L^−1^. There was a non-significant reduction in blood HCO_3_^¯^ between 75 and 90 min after SB + CHO (− 1.6 ± 1.7 mmol·L^−1^) and PLA + CHO (− 1.6 ± 1.3 mmol·L^−1^) ingestion (or after baseline blood draw < − 1.9 ± 1.6 mmol·L^−1^ >) due to a standard 10 min warm-up (which took place between minutes 77 and 87). The reduction seems to be a physiological response to exercise, however, rarely taken into account with respect to its impact on buffering potential of ingested SB. The maintenance of increased level of blood HCO_3_^¯^ after the first bout of WAnT may arise from the fact that the metabolic perturbations elicited by maximal all-out exercises lasting less than 30 s are distinct from those occurring during intense exercise lasting from ~ 30 s to 5 min^17^.

Jones et al.^15^ observed that 75 min after ingestion of 0.3, 0.2 or 0.1 g·kg^−1^_BM_ of SB, mean blood HCO_3_^¯^ was 31.7 ± 1.9, 30.1 ± 1.0, and 27.9 ± 1.3 mmol·L^−1^, while the mean blood HCO_3_^¯^ peaks occurred at 120 (~ 32.4 mmol·L^−1^), 90–105 (~ 30.5 mmol·L^−1^) and 105 (~ 28.1 mmol·L^−1^) min post-ingestion. In the study by Gough et al.^18^ the mean time to peak blood HCO_3_^¯^ after ingestion of 0.3 and 0.2 g·kg^−1^_BM_ of SB ranged from 87 to 89 and 77 to 83 min. In general, it could be stated that the higher the dose of SB, the higher the peak HCO_3_^¯^ and the longer the time to reach this peak. Both the aforementioned studies^15,18^ investigated resting kinetics of blood HCO_3_^¯^ after SB intake. However, little is known about the kinetics of blood HCO_3_^¯^ when doses higher than 0.3 g·kg^−1^_BM_ are ingested or protocols in which SB ingestion was combined with the intake of small amounts of CHO. Protocols which tested acute single SB doses equal to 0.4 g·kg^−1^_BM_ are relatively scarce^19–27^, and unlike our protocol, most of them provided the acute dose of 0.4 g·kg^−1^_BM_ split into smaller doses before exercise (e.g., 2 × 0.2 g·kg^−1^_BM_ ingested 90 and 20 min^23,25^ or 90 and 60 min^24^ pre-exercise^23–25^) or over 1 h period from starting until the end of SB ingestion^19^), and no information on blood HCO_3_^¯^ kinetics is given. It seems reasonable to verify, if higher doses of ingested SB would also result in a longer time of sustaining a desirable and effective blood HCO_3_^¯^ elevation. In these circumstances, peak blood HCO_3_^¯^ might not be perceived as a single time point, but as a time frame or ‘peak bicarbonate window’. Indeed, in this study, HCO_3_^¯^ was elevated and remained stable for a 30 min period. It has been previously shown that in a rested condition ingestion of 0.3 g·kg^−1^_BM_ of SB resulted in HCO_3_^¯^ increase > 6 mmol∙L^−1^ between 90 and 225 min^28^. Regarding practical applications, the individualized ingestion timing might be more important when implementing lower doses of SB (e.g., ≤ 0.2 g·kg^−1^_BM_). It is also worth mentioning, that when the HCO_3_^¯^ load exceeds the amount of acid in the stomach, HCO_3_^¯^ enters the intestine and reaches the jejunum, where it can be absorbed^11^. Thus, this phenomenon may partially explain the longer time to obtain peak HCO_3_^¯^ after consumption of high dose of SB compared to lower doses.

In the current study, SB was co-ingested with 15 g of CHO, which may have impacted the kinetics of blood HCO_3_^¯^. The addition of CHO was implemented based on previous literature, as a means to alleviate possible GI symptoms, and improving SB absorption. Carr et al.^29^, compared 8 different protocols of SB ingestion (0.3 g·kg^−1^_BM_), which differed in the form of supplement (solution or capsule), ingestion period (30 or 60 min), volume of fluid (7 or 14 mL·kg^−1^_BM_) and SB intake with a meal (containing 1.5 g CHO·kg^−1^_BM_). The most effective protocol for eliciting increases in blood HCO_3_^¯^ was SB supplementation in the form of capsules in conjunction with a CHO-rich meal, and when participants ingested the supplement within 30 min from commencement to completion of ingestion. Peak blood HCO_3_^¯^ (~ 30.9 mmol·L^−1^) was reached 150 min after ingestion, and the HCO_3_^¯^ elevation compared to baseline was ~  + 6.6 mmol·L^−1^. The analogous model which, however, did not include co-ingestion of the meal, resulted in achieving earlier (~ 120 min) peak blood HCO_3_^¯^ after SB intake. Thus, the co-ingestion of SB with CHO may delay reaching the peak of blood HCO_3_^¯^, and as a result separates the moment of peak HCO_3_^¯^ (which presumably refers to highest ergogenic effect) and the moment of peak GI symptoms (which results in performance decrements). However, the absolute dose of CHO provided in the current study was equal for each participant (15 g) and when expressed in relation to BM, was substantially lower than in the study by Carr et al.^29^. Thus, CHO impact on bicarbonate kinetics after ingestion of SB in the current study might be less pronounced compared to the study by Carr et al.^29^, and additionally affected by the warm-up which preceded the test exercise. Furthermore, in the current study, the GI side effects were evaluated only at a single time point, which was 120 min after SB + CHO or PLA + CHO intake. In the study by Gough et al.^18^, the time to peak GI upset was reached 36 and 29 min after ingestion of 0.3 and 0.2 g·kg^−1^_BM_. However, Carr et al.^29^, observed the greatest incidence of GI side effects 90 min after ingestion of 0.3 g·kg^−1^_BM_ SB in a solution form. On the one hand, the great discrepancy with regard to peak GI upset after ingestion of the same dose of SB suggests the need for more precise investigation of that aspect in the future studies. On the other hand, due to its subjective nature GI symptoms are difficult to measure accurately.

It is also worth considering that not the absolute value of peak blood HCO_3_^¯^, but the actual increase (change) in blood bicarbonate from baseline to peak HCO_3_^¯^, or to the moment exercise is initiated, might be the crucial factor for extra-cellular buffering support and the ergogenic efficiency of SB. According to a recent meta-analysis^16^, greater effects in exercise were obtained when blood bicarbonate increases were medium (4–6 mmol·L^−1^) and large (> 6 mmol·L^−1^) compared to small (≤ 4 mmol·L^−1^). In the current study, the mean increase in blood bicarbonate from baseline to blood HCO_3_^¯^ peak after SB + CHO ingestion was 7.8 ± 1.9 mmol·L^−1^; 16% of participants reached an increase in blood HCO_3_^¯^ of 4–6 mmol·L^−1^, which according to the previous literature corresponds to a zone of a potential ergogenic effect, and 84% of participants reached the increase of > 6 mmol·L^−1^, which corresponds to an almost certain ergogenic effect. However, what seems to be more important, is the mean increase of blood bicarbonate at 0′_EX_ which was 6.1 ± 1.9 mmol·L^−1^. In 9 participants (47.4%) the blood bicarbonate increase fell within the range of 4–6 mmol·L^−1^ at 0′_EX_ (90 min post-ingestion) and in the remaining 10 participants (52.6%) it was > 6 mmol·L^−1^. However, in the current study, in accordance with previous findings^30^ no significant relationships between HCO_3_^¯^ increase and WAnTs performance were observed. The lack of simultaneous data on blood HCO_3_^¯^ time to peak and/or peak concentration increase and time to peak GI symptoms upset, could be perceive as the limitation of the current study.

Based on the results of the current study and assuming that the greatest ergogenic potential of SB is noted when blood HCO_3_^¯^ reach maximum and the elevation is > 6 mmol·L^−1^, the implemented dosage of SB + CHO should have been ingested at least 75 min before the exercises started, or individualized ingestion times should be established based on individual blood HCO_3_^¯^ peak increases. However, it should also be emphasized, that the slight depletion in HCO_3_^¯^ concentration is unavoidable, as a consequence of the warm-up, which in turn is indispensable and essential to perform before staring competition or main training^31^. Furthermore, the exclusion of occurrence of GI side effects (impairing SB-induced exercise performance benefits) presumably requires extending the time between SB intake and exercise.

The ingestion of 0.4 g·kg^−1^_BM_ of SB + 15 g CHO ninety min before exercise did not prevent decrements in performance during consecutive bouts of 15-s WAnTs. To our knowledge, this the first study to implement such a demanding exercise protocol, comprising of 6 bouts of the WAnT with 30-s rest between bouts. In previous studies on SB supplementation, single^32^, two^33–35^, three^36–38^ or four^39–41^ bouts of 30-s WAnT were implemented. Similarly to our study, Zabala et al.^37^ found no impact of 0.3 g·kg^−1^_BM_ of SB 90 min before exercise on performance during 3 bouts of 30-s WAnT separated by 30-min or 15-min rest periods in elite cyclists. Only one study utilizing 4 bouts of the WAnT in judoists following acute SB supplementation (0.3 g·kg^−1^_BM_ of SB ingested 120 min before exercise) showed significantly higher AP (3rd and 4th bouts) and PP (4th bout) of upper limb WAnTs^39^. This SB supplementation timing could be entirely sufficient to avoid any GI disorders while effectively increasing the buffering capacity at the same time. This idea may be supported by the study by de Oliveira and colleagues^40^, where 5-day supplementation with 0.5 g·kg^−1^_BM_ of SB (split into four equal doses per day) resulted in the overall improvement (as measured by total mechanical work) in four bouts of upper limb WAnTs. The last dose of SB was ingested 4 h before the performance assessment. Similarly, Tobias et al.^41^ found that 7 days supplementation of 0.5 g·kg^−1^_BM_ of SB (no data of the last dose intake time before exercise) increased total work done in 4 bouts of 30-s upper-body WAnTs in combat sport athletes.

The undeniable and urgent need for controlling and quantifying the impact of GI side effects on performance after SB ingestion has been raised in two recent extensive papers on SB efficacy^11,16^. In general, the severity of GI side effects is dose-dependent, with higher doses resulting in more severe GI upset^11,21^, while studies indicate a variability in time to peak of GI upset^18,29^. Siegler et al.^42^ noted significant increases in the incidence of GI symptoms after ingestion of 0.3 g·kg^−1^_BM_ of SB at 30, 60, 90, 120 and 150 min, but not 180 min post-ingestion compared to baseline. Simultaneously, the individual susceptibility to GI side effects and their severity should be taken into thoughtful consideration. In the current study, based on the IQR of total GI symptoms score (Table 2), large variability in occurrence and severity of GI upset between study participants was observed. Furthermore, there were some participants whose total GI symptoms score was higher (*n* = 2) or equal (*n* = 1) in CTRL compared to SB + CHO. This suggests that there may be some individuals who exhibit high levels of anxiety, which naturally predisposes them to GI distress in laboratory or field-based conditions, regardless of supplementation or familiarization procedures. Saunders and colleagues^30^ found a significant overall performance improvement after SB ingestion only when excluding participants (4 individuals out of 21 participants) who experienced GI discomfort. In the current study, based on regression analysis, we found a significant adverse impact of GI symptoms severity on WAnT performance. Thus, the impact of GI distress must be taken into consideration when analyzing the ergogenic potential of SB, and particularly when protocol assuming implementation of (1) single (2) acute (3) high (≥ 0.4 g·kg^−1^_BM_) doses of SB. Highly-motivated elite athletes may overcome GI upset and still benefit from high doses of SB supplementation, though this should be experimentally confirmed.

One of the suggested strategies to minimize GI side effects after SB ingestion, is to ingest SB alongside a high-carbohydrate meal^11,29^. In the current study we combined SB and PLA ingestion with a small CHO (15 g) dose, in an attempt to maximize tolerance to the relatively high SB dose. Though the implemented strategy did not avoid GI symptoms, adopted protocol did not intend to provide greater amounts of CHO, to avoid achieving ergogenic effects arising from consumption of CHO. The source of CHO was an orange juice (150 mL) added to the tested preparations just before intake. It must to be mentioned, that the organic acids present in the juice (e.g., citric, ascorbic or malic) were available to react with H_2_O or NaHCO_3_. These weak organic acids partially dissociate in water. They react with sodium bicarbonate, meaning sodium salts, water and carbon dioxide are produced. Thus, one the hand, the addition of orange juice might slightly decrease the overall buffering potential of the implemented dose of SB. On the other hand, it improved substantially the taste of the preparations, and eventually might lead to greater palatability of supplementation. Moreover, the addition of the juice did not prevent from obtaining effective increase in blood bicarbonate.

One of the limitations of the current study is evaluation of the incidence and severity of GI symptoms at one solitary time point. Knowing the results of the study, and the fact that the severity of GI symptoms negatively impacted the WAnTs, it would be reasonable to evaluate GI symptoms at additional time points e.g., pre-supplementation and during exercise. This approach might help to identify the time of peak GI symptoms, and in the future studies to establish time of SB ingestion according to individual bicarbonate kinetics and GI symptoms occurrence. Another limitation of the study is that mean peak increase in blood HCO_3_^¯^ (7.8 ± 1.9 mmol·L^−1^) was achieved 15 min before the exercise test started. At the time point, right after 10 min standardized warm-up and before the exercise started (0′_EX_), the mean increase of blood HCO_3_^¯^ was just slightly above the threshold of almost certain ergogenic effect (~ 6.1 mmol·L^−1^). The strength of our study is a simultaneous comparison of blood bicarbonate kinetics and anaerobic performance. Most of the previous studies, which considered indices related to physical performance and high doses of SB (≥ 0.4 g·kg^−1^_BM_) controlled blood HCO_3_^¯^, lactate or pH (or other indices related to acid–base blood balance) solely at baseline, before and after exercise. In our study we insightfully controlled blood indices at 12 distinct time points. Thus, the current study must be perceived as highly innovative, and its undeniable strength is the ability to identify the peak blood HCO_3_^¯^, as well as peak increase (changes) in blood HCO_3_^¯^ and an increase (changes) in blood HCO_3_^¯^ directly before exercise was initiated, and to demonstrate there was a difference between these. Additionally, these blood collections clearly indicated the effect of warm-up causing a slight blood HCO_3_^¯^ depletion. The phenomenon is unavoidable, and has been raised up in one previous study^43^, in which similarly to current results, a warm-up (regardless its intensity) led to decrease in blood bicarbonate, but did not affect the ergogenic potential of ingested SB. That seems to be valuable in the perspective of the future studies, to control if the time of starting the exercise and time of reaching peak increase in blood HCO_3_^¯^ overlap and/or are located in the assumed and desirable time gap (e.g. in case of longer-lasting exercises). It is possible that in some previous studies the time for the exercise tests to initiate following supplementation was not optimal from the point of view of peak HCO_3_^¯^ increases in blood, which may affect conclusions. To the best of our knowledge, this is the first study to investigate the kinetics of blood bicarbonate (and other acid–base blood indices) after ingestion of a single acute dose of 0.4 g·kg^−1^_BM_ of SB. Finally, the undeniable strength of our study is also proving that to have a better insight into factors determining the buffering and ergogenic potential of SB, it is crucial to monitor blood HCO_3_^¯^ not only at baseline (pre-ingestion) and right before the exercise start, but also in between the ingestion and start of the exercise.

## Conclusions

In this study in CrossFit trained athletes, the acute ingestion of single dose of 0.4 g·kg^−1^_BM_ of sodium bicarbonate combined with a small amount of carbohydrate resulted in peak blood bicarbonate about 75 min after ingestion and significant increase in extra-cellular buffering capacity of plasma persisting from 60 until 90 min after intake. There was a significant negative impact of GI symptoms distress on mean indices of anaerobic performance of WAnT bouts. This may partially explain lack of improvement and/or lack of preserving from the decrements in performance in the six following bouts of the WAnT after SB ingestion. Thus, monitoring the time course of GI symptoms occurrence and severity post-SB intake seems to be crucial factors to control when implementing high, single, and acute doses of SB. Future studies should aim to investigate individualization of time of ingesting SB, combining the simultaneous elevation of blood bicarbonate referring to the zone of almost certain ergogenic action and lowest possible level of GI upset.

## Material and methods

### Participants

Twenty male and female CrossFit-trained practitioners were initially enrolled in this study and randomly allocated to the double-blinded treatment sequence (SB + CHO → PLA + CHO or PLA + CHO → SB + CHO) in a balanced design. There was one drop-out from the study, due to personal reasons (PLA + CHO → SB + CHO sequence). A total of 19 participants aged 37 ± 8 years and of mean body mass 75.1 ± 13.1 kg, completed the whole study protocol and were included in the analyses (Table 4).

**Table 4:** Baseline characteristics of studied group.

	All *n* = 19	SB + CHO → PLA + CHO *n* = 10	PLA + CHO → SB + CHO *n* = 9	*p*
Males (*n*)	9	5	4	–
Females (*n*)	10	5	5	–
Age (years)	37 ± 8	37 ± 5	37 ± 10	0.95
Height (m)	1.73 ± 0.09	1.74 ± 0.08	1.72 ± 0.10	0.58
Body mass (kg)	75.1 ± 13.1	74.9 ± 12.6	75.4 ± 14.5	0.87
Fat mass (%)	20.7 ± 6.7	20.7 ± 2.6	20.7 ± 9.7	0.99
Fat mass (kg)	15.7 ± 6.1	15.5 ± 2.8	15.9 ± 8.6	0.87
Fat free mass (kg)	59.5 ± 11.0	59.4 ± 10.5	59.5 ± 12.3	0.71
Total body water (kg)	39.5 ± 7.4	39.6 ± 7.5	39.5 ± 7.8	0.90
Training characteristics				
HIFT experience (years)	4.5 ± 1.3	4.5 ± 1.5	4.6 ± 1.2	0.65
Number of HIFT trainings per week (units)	4.1 ± 0.7	4.0 ± 0.6	4.3 ± 0.8	0.12
Duration of single HIFT training unit (min)	68 ± 12	70 ± 14	66 ± 11	0.54

Data are mean ± SD. *HIFT* high-intensity functional training.

The inclusion criteria were: good general health, a valid and up-to-date medical certificate that confirmed the athlete’s ability to practice sports, at least 4 years of training experience and participation in a minimum of four CrossFit workout sessions a week. The exclusion criteria were: current injury, health-related contraindication, declared general feeling of being unwell and unwillingness to follow the study protocol. The entire protocol for each participant lasted 4 weeks. All the study procedures were performed in the sport club in which participants practice CrossFit on regular basis (Lubin, Lubuskie Province, Poland).

**Figure 4:**
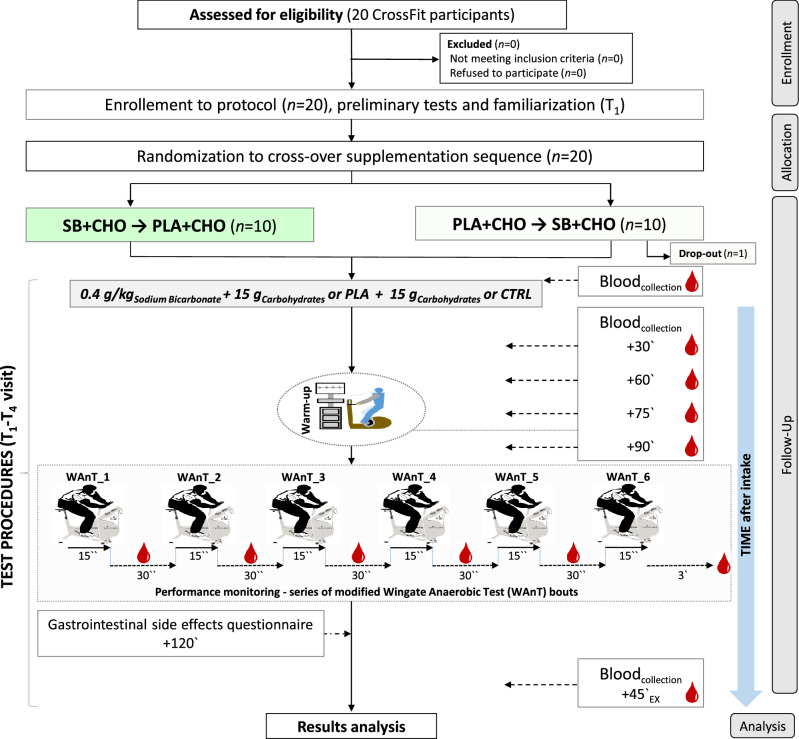
A flow chart of the study design.

The study protocol was reviewed and approved by the local institutional review board (Bioethics Committee at Poznan University of Medical Sciences, reference number: 1000/18 of 11 October 2018), and registered at ClinicalTrials.Gov (NCT03810404, 18/01/2019). Written informed consent was obtained from all participants before their participation in the study began. All procedures were conducted in accordance with the ethical standards of the 1975 Declaration of Helsinki. The study complies with the CONSORT statement for randomized trials, as shown in Fig. 4.

G*Power software (version 3.1.9.4, Universität Düsseldorf, Germany) was used to calculate the sample size required to obtain a power of approximately 80% (α = 0.05) and large effect size partial eta squared 0.14^44^ in analysis of variance (ANOVA) with repeated measurements (RM) within factors. Analysis indicated that a sample size of 12 would be suitable for detecting a difference between three measurements. To account for possible dropouts, 20 participants were initially enrolled into the study.

### Experimental protocol

In the current study, the efficacy of SB supplementation was evaluated in a randomized, cross-over, placebo-controlled double-blind trial. The protocol of the study consisted of four study visits (T_1_–T_4_) (Fig. 4), of which the T_1_ was arranged to obtain the anthropometric measurements of the participants and to get the participants familiarized with the study procedures, including the exercise protocol—several consecutive bouts of the 15 s WAnTs; the T_2_ was the control (CTRL) visit—no supplementation was provided, but all the testing procedures were performed; during the T_3_ and T_4_ visits the participants randomly ingested SB + CHO or placebo (PLA) + CHO. The T_2_–T_4_ visits were separated by 7-days washout period. Participants were obligated to eat a standardized meal two hours before each study visit. Furthermore, the T_2_–T_4_ visits started with BM and body composition evaluation, then the baseline blood samples (− 90′_EX_) were drawn, after which the participants ingested SB + CHO or PLA + CHO (T_3_ and T_4_ visits). Subsequent blood samples were taken 30 (− 60′_EX_), 60 (− 30′_EX_), 75 (− 15′_EX_) and 90 (0′_EX_) min after supplement ingestion, immediately after each of six bouts of WAnT (WAnT_1_POST_, WAnT_2_POST_, WAnT_3_POST_, WAnT_4_POST_, WAnT_5_POST_, WAnT_6_POST_), and the last sample was drawn during recovery − 45 min after completion of the whole exercise protocol (+ 45′_EX_). In total, 12 blood samples were taken from each participants during T_2_–T_4_ visits. At 120 min post-ingestion (in T_3_–T_4_) or at 120 min post-baseline blood draw (T_2_ at CTRL), participants completed a GI side effect questionnaire. Blood bicarbonate kinetics and performance in subsequent bouts of WAnT were considered as primary outcomes, while gastrointestinal side effects as secondary outcomes.

### Supplementation

The participants were provided with individually adjusted supplementation doses. The dose of supplemented SB was 0.4 g·kg^−1^_BM_ of SB + 15 g CHO. SB provided in the form of powder containing 89.1% of sodium bicarbonate, 9.9% of potassium bicarbonate, and 1.0% of sodium citrate (Alkala N, Sanum-Kehlbeck GmbH & Co. KG, Germany). The share of SB in the powder was taken into account when establishing individual supplemented doses. PLA preparation was provided in a similar powder form and contained sodium chloride in an equimolar amount of sodium as SB. Both preparations were dissolved in approximately 750 mL of fluid (600 mL of water and 150 mL of orange juice). Juice was provided as a source of CHO, which was supposed to alleviate GI symptoms^29^ and improve the taste of the preparations. Participants were asked to drink the preparations within 5 min. Neither main investigators, nor the participants were aware about the ingested preparations. The random allocation sequence and matching were performed using stratified randomization via impartial biostatistics, who was not directly involved in performing test procedures.

### Anthropometric measurements

Body mass and height were measured using a professional medical scale with a stadiometer (WPT 60/150 OW, RADWAG, Radom, Poland; an accuracy of 0.1 cm and 0.1 kg for height and BM). Body composition and total body water were assessed by means of bioelectric impedance, using Bodystat 1500 (Bodystat Inc., Douglas, UK).

### Blood samples analysis

Capillary blood was collected from a fingertip of the nondominant hand using a disposable lancet-spike Medlance Red (HTL-STREFA, Łódź, Poland) with a 1.5 mm blade and 2.0 mm penetration depth. Approximately 65 μL of blood was collected to a heparinized capillary tube where HCO_3_^−^, H^+^, Na^+^, K^+^, Cl^−^, base excess, anion gap, glucose, and lactate concentrations and pH value were determined on blood gas analyzer (ABL90 FLEX, Radiometer, Brønshøj, Denmark). The blood gas analyzer was each time calibrated before use.

### Power indices monitoring—series of modified Wingate Anaerobic Test (WAnT) bouts

Six bouts of the 15 s WAnT on a cycloergometer (Monark 894E, Varberg, Sweden) were performed with 30 s rests between consecutive bouts. The recommendations proposed by Bar-Or ^45^ were followed, as described in our previous studies^33,34^. The following indices were evaluated: peak power (PP), average power (AP), minimum power (MP), power drop (PD), and time to PP (T_to_PP). The series of six bouts of 15 s WAnTs was preceded by a standardized 10 min warm-up (from 77 until 87 min after SB + CHO or PLA + CHO ingestion or baseline blood drawn < CTRL >), of which the first 5 min were cycling at approximately 50 W followed by 5 min of self-selected stretching.

### Gastrointestinal side effects

GI side effects were evaluated via a standardized questionnaire^46^, which has been previously adapted and effectively utilized in our former studies^9,33^ and studies on SB supplementation by others^18,29^. The questionnaire consists of 19 items describing common GI symptoms and the numeric rating scale (NRS) was used to rate the intensity of these symptoms. NRS ranged between 0 and 10, where 0 reflected no GI distress at all and 10 being the most severe GI distress imaginable. The total GI score was calculated as a sum of all 19 GI symptoms. GI symptoms were considered severe when a score ≥ 5 was given, and less severe when < 5. The exception was vomiting, which was considered severe when scores were > 0. The severe GI symptoms included nausea, stomach ache, urge to vomit, vomiting and intestinal cramps. Less severe GI symptoms included eructation, flatulence, urge to defecate, heartburn and abdominal pressure (bloating). The sum of scores was then calculated for severe and less severe symptoms^46^.

### Statistical analysis

All variables were checked for normal distribution using the Shapiro–Wilk test. Blood variables and the WAnTs are presented as mean ± 1 standard deviation (SD); 95% confidence interval (95% CI). Baseline comparison between subgroups according to supplementation sequence were performed using *T*-test for independent samples or Mann–Whitney *U*-test. Blood variables between study conditions (CTRL, SB + CHO, PLA + CHO) at each of twelve particular time points (from − 90′_EX_ to + 45′_EX_), were tested with repeated-measures analysis of variance (RM ANOVA). Similarly, the results of the WAnTs (the differences between six consecutive bouts from WAnT_1 to WAnT_6) between the study conditions (CTRL, SB + CHO or PLA + CHO) were analyzed via RM ANOVA. For the WAnT (performed six times on each testing day at each of the three study conditions—CTRL, SB + CHO, PLA + CHO) the three different models of RM ANOVA were used, of which the first one was univariate model (^†^bouts *x* treatment) and the two remaining were double-multivariate models (^ǂ^bouts *x* treatment *x* treatment order or ^§^bouts *x* treatment *x* gender). Taking into account the robustness of *F*-test in terms of Type 1 error^47^, if the normality assumptions based on Shapiro–Wilk test was violated, the kurtosis and skewness variables were also evaluated. Eventually, all the variables met the assumptions to be analyzed by various models of ANOVA. A Huynh–Feldt adjustment was made when sphericity was violated (as indicated by Mauchly’s test). Post-hoc comparisons were performed by Bonferroni test. Effect sizes are expressed as partial eta square (*η*^*2*^_*p*_). The results of GI side effects are presented as median ± interquartile range (IQR); quartile range. The results were analyzed by Friedman’s ANOVA, followed by post-hoc for Friedman. However, the power of post-hoc for Friedman was not always strong enough to detect the substantial differences between the compared groups (i.e., nausea, dizziness, urge to urinate, bloating, cold shivering), even though the Friedman’s ANOVA indicated their existence. Effect sizes were calculated using Kendall’s concordance coefficient *W* (0—no agreement and 1—complete agreement). The relationship between GI symptoms (total GI symptoms score, severe GI symptoms and less severe GI symptoms) and WAnT performance (the mean results of six WAnT bouts), as well as the relationship between the increase in blood bicarbonate and performance in particular bouts of WAnT were analyzed using Spearman's rank correlation. Statistical significance was set at *p* < 0.05. The different letter inscriptions in the results presentation indicate substantial differences between measurements/study conditions. Data were analyzed using STATISTICA 13.3 (StatSoft Inc., Tulsa, OK, USA) software.

## Supplementary Information

Supplementary Information.

## Data Availability

The original contributions presented in the study are included in the article/supplementary material, further inquiries can be directed to the corresponding author.
