# Supplementary material for: The interplay between bicarbonate kinetics and gastrointestinal upset on ergogenic potential after sodium bicarbonate intake: a randomized double-blind placebo-controlled trial
Source: Sci Rep. 2023 May 1;13:7081. doi: 10.1038/s41598-023-34343-0 (PMC10151363; doi:10.1038/s41598-023-34343-0)
Supplement: Supplementary file 1 — Supplementary Information. [file 41598_2023_34343_MOESM1_ESM.docx]

The Interplay Between Bicarbonate Kinetics and Gastrointestinal Upset on Ergogenic Potential after Sodium Bicarbonate Intake

Krzysztof Durkalec-Michalski^1,2,*^, Paulina M. Nowaczyk^1^, Joanna Kamińska^3^, Bryan Saunders^4,5^, Igor Łoniewski^6^, Dominika Czubaszek^1^, Michal Steffl^2^, Tomasz Podgórski^3^

^1^Department of Sports Dietetics, Poznan University of Physical Education, Poznan, Poland

^2^Department of Physiology and Biochemistry, Faculty of Physical Education and Sport, Charles University, Prague, Czech Republic

^3^Department of Physiology and Biochemistry, Poznan University of Physical Education, Poznan, Poland

^4^Applied Physiology and Nutrition Research Group, School of Physical Education and Sport, Rheumatology Division, Faculdade de Medicina FMUSP, University of São Paulo, São Paulo, BRAZIL

^5^Institute of Orthopedics and Traumatology, Faculty of Medicine FMUSP, University of São Paulo, Brazil

^6^Department of Biochemical Sciences, Pomeranian Medical University in Szczecin, Szczecin, Poland

*** Correspondence:**Corresponding Author

Krzysztof Durkalec-Michalski

Department of Sports Dietetics, Poznan University of Physical Education, 61-871 Poznan, Poland

Tel.: +48 61 835 51 65

e-mail: [durkalec-michalski@awf.poznan.pl](mailto:durkalec-michalski@awf.poznan.pl)

**Supplementary table S1.** Blood biochemical markers results in CTRL, SB+CHO and PLA+CHO conditions.

| **Indicator** | **Condition** |  | **Baseline**  **-90’_EX_** | **30 min post-ingestion**  **-60’_EX_** | **60 min post-ingestion**  **-30^’^_EX_** | **70 min post-ingestion**  **-15^’^_EX_** | **90 min post-ingestion**  **0^’^_EX_** | **WAnT_1_POST_** | **WAnT_2_POST_** | **WAnT_3_POST_** | **WAnT_4_POST_** | **WAnT_5_POST_** | **WAnT_6_POST_** | **45 min after EX** |
| --- | --- | --- | --- | --- | --- | --- | --- | --- | --- | --- | --- | --- | --- | --- |
| **H^+^** | CTRL  SB+CHO  PLA+CHO | (nmol·L^-1^)  (95%CI)  (nmol·L^-1^)  (95%CI)  (nmol·L^-1^)  (95%CI)  *p* | 38.0 ± 1.6  37.2 – 38.8  38.7 ± 2.0  37.8 – 39.7  38.4 ± 1.4  37.8 – 39.1  0.271 | 38.5 ± 1.5^b^  37.8 – 39.2  34.6 ± 1.9^a^  33.7 – 35.6  39.3 ± 1.6^b^  38.5 – 40.1  0.000 | 38.0 ± 1.5^b^  37.2 – 38.7  33.3 ± 3.1^a^  31.9 – 34.8  39.8 ± 2.0^c^  38.9 – 40.8  0.000 | 37.6 ± 1.6  36.8 – 38.4  32.1 ± 2.0  31.1 – 33.1  40.8 ± 2.0  39.8 – 41.8  0.000 | 39.4 ± 2.3^b^  38.3 – 40.5  32.6 ± 2.5^a^  31.4 – 33.8  42.6 ± 2.5^c^  41.4 – 43.9  0.000 | 39.6 ± 3.6^b^  37.9 – 41.4  31.5 ± 2.9^a^  30.1 – 33.0  41.7 ± 4.2^b^  39.7 – 43.8  0.000 | 45.0 ± 2.5^b^  43.8 – 46.3  37.5 ± 2.0^a^  36.5 – 38.4  50.2 ± 2.8^c^  48.8 – 51.5  0.000 | 52.6 ± 4.2^b^  50.6 – 54.6  43.0 ± 2.4^a^  41.9 – 44.2  58.4 ± 4.1^c^  56.5 ± 60.4  0.000 | 60.8 ± 6.2^b^  57.8 – 63.8  48.9 ± 2.9^a^  47.6 – 50.3  68.2 ± 8.1^c^  64.3 – 72.1  0.000 | 67.1 ± 7.0^b^  63.8 – 70.5  53.8 ± 4.5^a^  51.7 – 56.0  74.2 ± 8.1^b^  70.4 – 78.1  0.000 | 83.3 ± 12.7^b^  77.1 – 89.4  67.7 ± 12.0^a^  61.9 – 73.5  91.7 ±10.8^c^  86.5 ± 96.9  0.000 | 44.3 ± 5.0^b^  41.9 – 46.7  34.5 ± 4.2^a^  32.4 – 36.5  46.3 ± 3.9^b^  44.4 – 48.2  0.000 |
| **Base**  **excess** | CTRL  SB+CHO  PLA+CHO | (mmol·L^-1^)  (95%CI)  (mmol·L^-1^)  (95%CI)  (mmol·L^-1^)  (95%CI)  *p* | 2.1 ± 1.5^b^  1.4 – 2.8  1.4 ± 1.6^ab^  0.7 – 2.2  1.2 – 2.0^a^  0.2 – 2.2  0.015 | 1.5 ± 1.6^a^  0.7 – 2.2  6.0 ± 2.3^b^  4.9 – 7.1  0.9 ± 1.7^a^  0.1 – 1.8  0.000 | 1.9 ± 1.7^b^  1.1 – 2.7  9.4 ± 3.1^c^  7.9 – 10.9  0.63 ± 1.56^a^  -0.13 – 1.38  0.000 | 2.0 ± 1.8^b^  1.2 – 2.9  10.7 ± 2.9^c^  9.3 – 12.1  0.03 ± 1.73^a^  -0.80 – 0.87  0.000 | -0.5 ± 1.6^b^  -1.3 – 0.3  8.7 ± 2.5^c^  7.5 – 9.9  -2.4 ± 2.1^a^  -3.4 – -1.3  0.000 | -1.5 ± 3.1^a^  -3.0 – -0.0  9.1 ± 2.8^b^  7.8 – 10.5  -2.4 ± 2.2^a^  -3.4 – -1.3  0.000 | -5.3 ± 2.3^b^  -6.4 – -4.2  3.0 ± 2.3^c^  1.9 – 4.1  -7.4 ± 2.1^a^  -8.4 – -6.4  0.000 | -9.6 ± 2.2^b^  -10.7 – -8.6  -2.3 ± 1.9^c^  -3.2 – -1.3  -11.6 ± 2.3^a^  -12.7 – -10.5  0.000 | -13.3 ± 2.8^b^  -14.6 – -11.9  -6.5 ± 2.0^a^  -7.5 – -5.6  -15.4 ± 3.1^c^  -16.9 – -13.9  0.000 | -15.8 ± 2.6^b^  -17.0 – -14.5  -9.6 ± 2.1^c^  -10.5 – -8.6  -17.7 ± 2.5^a^  -18.9 – -16.5  0.000 | -21.1 ± 2.9^b^  -22.5 – -19.8  -16.6 ± 3.2^c^  -18.1 – -15.0  -22.7 ± 2.4^a^  -23.9 – -21.6  0.000 | -5.7 ± 3.5^a^  -7.4 – -4.1  4.2 ± 5.8^b^  1.4 – 7.0  -7.0 ± 3.1^a^  -8.5 – -5.5  0.000 |
| **Na^+^** | CTRL  SB+CHO  PLA+CHO | (mmol·L^-1^)  (95%CI)  (mmol·L^-1^)  (95%CI)  (mmol·L^-1^)  (95%CI)  *p* | 143 ± 1^b^  142 – 143  141 ± 1  141 – 142^a^  142 ± 2  141 – 143^ab^  0.008 | 141 ± 2^a^  140 – 142  145 ± 1^b^  145 – 146  145 ± 2^b^  144 – 146  0.000 | 141 ± 1^a^  140 – 141  147 – 1^b^  146 – 148  146 – 1^b^  146 – 147  0.000 | 141 ± 1^a^  140 – 141  148 ± 2^c^  147 – 148  147 ± 2^b^  146 – 147  0.000 | 141 ± 2^a^  140 – 142  147 ± 2  146 – 148^c^  146 ± 2  145 – 147^b^  0.000 | 144 ± 6  141 – 147  149 ± 2  148 – 149  152 ± 19  143 – 161  0.162 | 144 ± 2^a^  143 – 145  150 ± 3^b^  149 – 152  149 ± 2^b^  148 – 151  0.000 | 146 ± 0.12^a^  145 – 147  152 ± 2^ab^  151 – 153  155 ± 16^b^  147 – 162  0.041 | 147 ± 2^a^  146 – 148  153 ± 3^b^  152 – 154  153 ± 2^b^  152 – 154  0.000 | 151 ± 11  145 – 156  154 ± 2  153 – 155  154 ± 2  153 – 155  0.195 | 145 ± 3^a^  144 – 147  151 ± 3^b^  150 – 153  151 ± 2^b^  150 – 153  0.000 | 140 ± 2^a^  139 – 140  145 ± 2  144 – 146^b^  145 ± 2  144 – 146^b^  0.000 |
| **K^+^** | CTRL  SB+CHO  PLA+CHO | (mmol·L^-1^)  (95%CI)  (mmol·L^-1^)  (95%CI)  (mmol·L^-1^)  (95%CI)  *p* | 4.3 ± 0.3  4.2 – 4.5  4.4 ± 0.2  4.2 – 4.5  4.4 ± 0.3  4.3 – 4.5  0.753 | 4.3 ± 0.3^b^  4.2 – 4.5  4.4 ± 0.2^b^  4.3 – 4.5  4.2 ± 0.3^a^  4.0 – 4.3  0.010 | 4.4 ± 0.2  4.3 – 4.5  4.4 ± 0.2  4.3 – 4.5  4.4 ± 0.5  4.2 – 4.7  0.979 | 4.5 ± 0.3  4.3 – 4.6  4.5 ± 0.3  4.4 – 4.6  4.4 ± 0.2  4.3 – 4.5  0.311 | 4.6 ± 0.03  4.4 – 4.7  4.4 ± 0.3  4.3 – 4.5  4.5 ± 0.3  4.4 – 4.6  0.271 | 5.3 ± 0.7  5.0 – 5.7  5.1 ± 0.4  4.9 – 5.3  5.4 ± 1.1  4.9 – 5.9  0.450 | 5.4 ± 0.5  5.1 – 5.6  5.4 ± 0.6  5.1 – 5.7  5.5 ± 0.5  5.2 – 5.7  0.745 | 5.7 ± 0.4  5.5 – 5.9  5.5 ± 0.5  5.3 – 5.8  5.5 ± 0.5  5.3 – 5.8  0.118 | 5.7 ± 0.5  5.5 – 6.0  5.6 ± 0.5  5.4 – 5.8  5.7 ± 0.6  5.4 – 6.0  0.638 | 5.8 ± 0.6  5.5 – 6.2  6.0 ± 1.9  5.1 – 6.9  5.8 ± 0.5  5.6 – 6.1  0.765 | 4.1 ± 0.4^b^  3.9 – 4.3  3.8 ± 0.3^a^  3.6 – 3.9  4.0 ± 0.^3ab^  3.9 – 4.1  0.006 | 4.2 ± 0.3^b^  4.1 – 4.4  4.0 ± 0.2^a^  3.9 – 4.1  4.5 ± 0.3^c^  4.3 – 4.6  0.000 |
| **Cl^-^** | CTRL  SB+CHO  PLA+CHO | (mmol·L^-1^)  (95%CI)  (mmol·L^-1^)  (95%CI)  (mmol·L^-1^)  (95%CI )  *p* | 106 ± 2  105 – 107  106 ± 2  105 – 107  107 ± 2  106 – 108  0.040 | 105 ± 2^a^  104 – 106  105 ± 2^a^  104 – 106  110 ± 2^b^  108 – 111  0.000 | 105 ± 2^a^  104 – 106  104 ± 3^a^  102 – 105  112 ± 3^b^  110 – 113  0.000 | 104 ± 2  103 – 105  103 ± 3  101 – 104  112 ± 3  111 – 114  0.000 | 105 ± 2^b^  103 – 106  102 ± 3^a^  101 – 103  113 ± 3^c^  111 – 115  0.000 | 107 ± 3^b^  105 – 108  103 ± 3^a^  101 – 104  115 ± 5^c^  113 – 117  0.000 | 108 ± 3^b^  107 – 109  105 ± 3  103 – 106^a^  116 ± 3^c^  114 – 118  0.000 | 110 ± 3^b^  108 – 112  106 ± 3^a^  105 – 107  117 ± 4^c^  116 – 119  0.000 | 110 ± 2^b^  109 – 111  107 ± 3  105 – 108^a^  118 ± 3^c^  117 – 120  0.000 | 111 ± 2^b^  109 – 112  107 ± 3^a^  106 – 108  119 ± 3^c^  117 – 120  0.000 | 108 ± 3^b^  106 – 109  104 ± 3^a^  103 – 105  116 ± 3^c^  114 – 117  0.000 | 104 ± 2^b^  103 – 105  100 ± 3^a^  98 – 102  112 ± 3^c^  111 – 113  0.000 |
| **Anion** | CTRL  SB+CHO  PLA+CHO | (mmol·L^-1^)  (95%CI)  (mmol·L^-1^)  (95%CI)  (mmol·L^-1^)  (95%CI)  *p* | 9.7 ± 1.2^b^  9.2 – 10.3  9.2 ± 1.3^ab^  8.6 – 9.8  9.0 ± 1.3^a^  8.4 – 9.7  0.037 | 9.8 ± 1.0  9.3 – 10.2  10.4 ± 1.3  9.7 – 11.0  9.8 ± 1.4  9.1 – 10.4  0.035 | 9.7 ± 1.0^a^  9.2 – 10.1  10.6 ± 1.4^b^  9.9 – 11.3  9.4 ± 1.9^a^  8.5 – 10.4  0.003 | 10.1 ± 1.2^ab^  9.5 – 10.7  10.9 ± 1.6^b^  10.2 – 11.7  9.3 ± 1.5^a^  8.6 – 10.0  0.000 | 12.2 ± 2.2^b^  11.1 – 13.2  13.2 ± 2.5^b^  12.0 – 14.4  10.4 ± 2.4^a^  9.2 – 11.5  0.000 | 14.1 ± 6.6^b^  10.9 – 17.3  13.3 ± 1.4^ab^  12.6 – 14.0  10.6 ± 1.9^a^  9.6 – 11.5  0.037 | 15.7 ± 1.9^a^  14.8 – 16.6  18.0 ± 2.3^b^  16.9 – 19.1  14.5 ± 2.3^a^  13.3 – 15.6  0.000 | 18.9 ± 2.8^a^  17.6 – 20.3  22.6 ± 2.1^b^  21.6 – 23.6  17.9 ± 2.1^a^  16.9 – 18.9  0.000 | 22.4 ± 2.9^a^  21.1 – 23.8  26.7 ± 2.5^b^  25.5 – 27.9  21.7 ± 3.0^a^  20.3 – 23.1  0.000 | 24.6 ± 2.3^a^  23.5 – 25.8  29.5 ± 2.4^b^  28.4 – 30.7  23.5 ± 2.8^a^  22.2 – 24.9  0.000 | 29.0 ± 2.6^a^  27.8 – 30.3  35.5 ± 3.4^b^  33.8 – 37.1  27.9 ± 2.9^a^  26.5 – 29.3  0.000 | 15.6 ± 3.7^ab^  13.8 – 17.4  17.4 ± 4.4^b^  15.2 – 19.5  14.1 ± 3.6^a^  12.4 – 15.9  0.008 |
| **Glucose** | CTRL  SB+CHO  PLA+CHO | (mg·dL^-1^)  (95%CI)  (mg·dL^-1^)  (95%CI)  (mg·dL^-1^)  (95%CI)  *p* | 104 ± 13  98 – 111  104 ± 12  98 – 110  102 ± 8  98 – 106  0.690 | 116 ± 12^b^  111 – 122  100 ± 10^a^  96 – 105  102 ± 6^a^  99 – 105  0.000 | 98 ± 9  94 – 102  97 ± 12  91 – 103  96 ± 9  92 – 101  0.893 | 97 ± 7^a^  93 – 100  103 ± 9^b^  99 – 107  97 ± 7^a^  94 – 101  0.010 | 96 ± 8^a^  93 – 100  102 ± 10^b^  97 – 106  95 ± 6^a^  92 – 98  0.008 | 100 ± 7^ab^  96 – 103  104 ± 9^b^  100 – 109  96 ± 8  92 – 100^a^  0.000 | 98 ± 7^a^  95 – 102  104 ± 9^b^  99 – 108  97 ± 8^a^  94 – 101  0.006 | 99 ± 10^a^  94 – 103  104 ± 9^b^  100 – 109  100 ± 8^ab^  96 – 104  0.040 | 101 ± 9  97 – 106  106 ± 9  101 – 110  102 ± 12  97 – 108  0.236 | 106 ± 10  101 – 110  107 ± 7  104 – 111  105 ± 7  101 – 108  0.437 | 140 ± 17^ab^  132 – 149  144 ± 22^b^  133 – 154  133 ± 18^a^  125 – 142  0.003 | 101 ± 20^ab^  92 – 111  105 ± 19^b^  96 – 115  93 ± 10^a^  88 – 98  0.003 |

Note: Data are presented as mean ± SD, 95% CI. ^a, b, c^ – different letters refers to significant differences between study conditions.

**Supplementary table S2**. Regression between performance in WAnTs and Δ in blood bicarbonate changes between baseline and peak bicarbonate or 90 min after intake (at time point of exercise start <0’_EX_>) after SB+CHO treatment

| **WAnT bouts** |  |  | **Peak power** | **Average power** | **Minimum power** | **Power drop** | **Time to peak power** |
| --- | --- | --- | --- | --- | --- | --- | --- |
| **WAnT_1** | Δ bicarbonate from baseline  to peak increase  Δ bicarbonate from baseline  to 90 min after intake (and at 0’_EX_) | *p*  *r*  *p*  *r* | 0.623  -0.124  0.314  -0.244 | 0.388  -0.217  0.184  -0.319 | 0.276  -0.271  0.258  -0.273 | 0.846  -0.049  0.297  -0.253 | 0.762  -0.077  0.960  -0.012 |
| **WAnT_2** | Δ bicarbonate from baseline  to peak increase  Δ bicarbonate from baseline to  90 min after intake (and at 0’_EX_) | *p*  *r*  *p*  *r* | 0.329  -0.244  0.632  -0.118 | 0.558  -0.148  0.864  -0.042 | 0.666  0.109  0.647  0.112 | 0.203  -0.315  0.208  -0.303 | 0.903  -0.031  0.710  -0.091 |
| **WAnT_3** | Δ bicarbonate from baseline  to peak increase  Δ bicarbonate from baseline to  90 min after intake (and at 0’_EX_) | *p*  *r*  *p*  *r* | 0.556  -0.149  0.647  -0.112 | 0.718  -0.091  0.606  -0.126 | 0.427  -0.200  0.745  -0.080 | 0.815  -0.060  0.622  -0.121 | 0.396  -0.213  0.393  -0.208 |
| **WAnT_4** | Δ bicarbonate from baseline  to peak increase  Δ bicarbonate from baseline to  90 min after intake (and at 0’_EX_) | *p*  *r*  *p*  *r* | 0.461  -0.186  0.383  -0.212 | 0.770  -0.741  0.814  -0.058 | 0.842  -0.050  0.566  0.140 | 0.483  -0.177  0.098  -0.391 | 0.940  -0.019  0.514  0.160 |
| **WAnT_5** | Δ bicarbonate from baseline  to peak increase  Δ bicarbonate from baseline to  90 min after intake (and at 0’_EX_) | *p*  *r*  *p*  *r* | 0.431  -0.198  0.318  -0.242 | 0.679  -0.105  0.589  -0.133 | 0.778  0.071  0.762  0.075 | 0.428  -0.199  0.363  -0.221 | 0.755  0.079  0.637  0.116 |
| **WAnT_6** | Δ bicarbonate from baseline  to peak increase  Δ bicarbonate from baseline to  90 min after intake (and at 0’_EX_) | *p*  *r*  *p*  *r* | 0.364  -0.228  0.789  -0.066 | 0.966  -0.011  0.878  0.038 | 0.772  0.074  0.743  -0.081 | 0.126  -0.374  0.514  -0.160 | 0.713  0.093  0.710  0.091 |
